# Adipose-Derived Stem Cells from Systemic Sclerosis Patients Maintain Pro-Angiogenic and Antifibrotic Paracrine Effects In Vitro

**DOI:** 10.3390/jcm8111979

**Published:** 2019-11-14

**Authors:** Mélanie VELIER, Stéphanie SIMONCINI, Maxime ABELLAN, Pauline FRANCOIS, Sandy EAP, Anaïs LAGRANGE, Baptiste BERTRAND, Aurélie DAUMAS, Brigitte GRANEL, Bruno DELORME, Françoise DIGNAT GEORGE, Jérémy MAGALON, Florence SABATIER

**Affiliations:** 1 Aix Marseille University, INSERM, INRA, C2VN, 13005 Marseille, France; 2 Cell Therapy Department, Hôpital de la Conception, AP-HM, INSERM CIC BT 1409, 13005 Marseille, France; 3 Plastic Surgery Department, Hôpital de la Conception, AP-HM, 13005 Marseille, France; 4 R&D Department, Macopharma, 59420 Mouvaux, France; 5 Internal Medicine Department, Hôpital Nord & Hôpital de la Timone, AP-HM, 13005 Marseille, France

**Keywords:** systemic sclerosis, adipose derived stromal/stem cells, paracrine effect, angiogenesis, antifibrotic

## Abstract

Innovative therapies based on autologous adipose-derived stem/stromal cells (ASC) are currently being evaluated for treatment of systemic sclerosis (SSc). Although paracrine angiogenic and antifibrotic effects are considered the predominant mechanisms of ASC therapeutic potential, the impact of SSc on ASC paracrine functions remains controversial. In this study, phenotype, senescence, differentiation potential, and molecular profile were determined in ASC from SSc patients (SSc-ASC) (*n* = 7) and healthy donors (HD-ASC) (*n* = 7). ASC were co-cultured in indirect models with dermal fibroblasts (DF) from SSc patients or endothelial cells to assess their pro-angiogenic and antifibrotic paracrine effects. The angiogenic activity of endothelial cells was measured in vitro using tube formation and spheroid assays. DF collagen and alpha smooth muscle actin (αSMA) content were quantified after five days of co-culture with ASC. Differentiation capacity, senescence, and mRNA profiles did not differ significantly between SSc-ASC and HD-ASC. SSc-ASC retained the ability to stimulate angiogenesis through paracrine mechanisms; however, functional assays revealed reduced potential compared to HD-ASC. DF fibrosis markers were significantly decreased after co-culture with SSc-ASC. Together, these results indicate that SSc effects do not significantly compromise the angiogenic and the antifibrotic paracrine properties of ASC, thereby supporting further development of ASC-based autologous therapies for SSc treatment.

## 1. Introduction

Systemic sclerosis (SSc) is a rare and multifaceted systemic autoimmune disease. Although SSc pathogenesis remains elusive, it is generally accepted that initial vascular injury due to autoimmunity can result in constitutive activation of fibroblasts and fibrosis affecting skin and multiple organs. The interaction of these three processes results in a polymorphous spectrum of clinical and pathologic manifestations of SSc [1]. In rapidly progressive SSc forms, mortality rates reach 30–50% within the first five years of disease onset depending on the extent of skin, the cardiopulmonary, and the renal involvement [2]. Current therapeutic approaches are insufficient to halt the evolution of the disease and cure fibrosis lesions that have already formed. To date, no treatment has offered any benefit to patients with severe forms of SSc, with the exception of autologous hematopoietic stem cell transplantation, which significantly reduces mortality but displays high toxicity [3,4,5]. In this context, innovative cell-based therapies are currently being developed in the hope that they may confer long-term benefits with higher tolerance. Mesenchymal stem/stromal cells (MSC) are multipotent progenitors that can be isolated from several tissues including bone marrow, adipose, umbilical cord, or placenta tissues [6]. The discovery of MSC’s trophic and immunomodulatory properties has accelerated the development of MSC-based therapies in regenerative medicine and for the treatment of autoimmune diseases [7]. MSC also exhibit antifibrotic [8] and angiogenic [9] capacities, which may counteract the main pathogenic mechanisms of SSc. MSC therapeutic potential has largely been attributed to paracrine activity. Soluble mediators and extracellular vesicles produced by MSC play a pivotal role in immunomodulation and tissue repair [10]. Adipose tissue MSC are abundant and easy to harvest, and adipose-derived stromal/stem cells (ASC) have been assessed in several SSc models in vivo with promising results. Three studies tested ASC in a bleomycin-induced SSc model and showed a reduction or prevention of lung fibrosis [11,12,13]. ASC from healthy donors (HDs) have been shown to reduce fibrotic and inflammatory markers and promote matrix remodeling in skin and lungs using a hypochlorous acid (HOCl) murine model of diffuse SSc [14]. 

Early phase clinical trials evaluating local injections of autologous ASC in the faces or the hands of SSc patients have yielded encouraging results. Transplantation of ASC in a hyaluronic acid solution significantly improved tightened skin [15]. Another study compared fat grafting and injection of autologous ASC in the mouths of SSc patients with functional disability and obtained satisfactory results with both procedures in terms of mouth opening and pain reduction [16]. Our research group reported the safety and the efficacy of an autologous adipose-derived stromal vascular fraction composed mainly of uncultured ASC in treating hand disability and Raynaud syndrome among SSc patients [17,18]. However, autologous MSC-based therapies remain controversial [19,20,21,22], notably concerning potential alteration of cells from SSc patients. Although most of these studies have focused on bone-marrow-derived MSC, ASC-based therapies are also controversial. Recent reports of altered molecular signatures in ASC from SSc patients have suggested compromise of their angiogenic and ant-fibrotic activity [23,24,25]; however, few functional studies have been conducted. 

Since paracrine effects are considered the predominant mechanism mediating therapeutic activity in ASC, the aim of this study was to investigate the extent to which SSc affects paracrine angiogenic and antifibrotic effects in ASC.

## 2. Experimental

### 2.1. Donor Specifications

ASC from healthy donors (HD-ASC; *n* = 7) were obtained from adipose tissue surgical residues following liposuction for aesthetic purposes. ASC from SSc patients (SSc-ASC; *n* = 7) were obtained from adipose tissue surgical residues following routine care lipotransfer. All patients and HD provided informed consent for the scientific use of surgical residues.

### 2.2. ASC Isolation and Expansion 

Adipose tissue harvesting was performed using a standardized method in a closed circuit with a 3 mm cannula (Coleman). Upon the completion of harvesting, the bag was immediately transported to a registered cell-therapy unit. The collected lipoaspirate was washed with Ringer’s lactate solution (Baxter Inc., Opelika, AL, USA) and enzymatically digested with 0.25 U/mL collagenase NB 5 (SERVA) for 45 min at 37 °C. Cells were concentrated, washed, aseptically recovered, and resuspended in Ringer’s lactate solution. The cell suspension was then plated into a T75 flask in Dulbecco’s Modified Eagle’s Medium and Ham’s F-12 Nutrient Mixture (DMEM/F-12) supplemented with 10% fetal bovine serum (FBS) and antibiotic solution. The culture medium was changed every 48 h until 80% confluence was reached. Adherent cells were then detached with trypsin-EDTA and passaged to extend the culture. ASC from passages 3–5 were used for all experiments.

### 2.3. Phenotypic Analysis

The expression of a panel of surface markers was assessed following previously described protocols (26). For each antigen, 200,000 cells resuspended in cold phosphate-buffered saline (PBS) were incubated with phycoerythrin (PE)-conjugated monoclonal antibody at saturating concentration for 30 min in the dark at 4 °C. Antibodies and isotype controls were purchased from Becton-Dickinson (CD29, CD34, CD40, CD44, CD45, CD73, CD90, CD105, IgG1, and IgG2b) or Beckman Coulter (CD80, CD86, and HLA-DR). Appropriate PE-conjugated isotype-matched controls were included. Cells were then washed twice with PBS Ca++/Mg++ by centrifugation at 300 *g* for 5 min. Labeled HD-ASC and SSc-ASC were finally resuspended in 200 μL CellFix (Becton-Dickinson) and processed immediately for flow cytometric analysis. Acquisitions were performed using an ACCURI C6 flow cytometer equipped with 488 nm argon laser (Becton-Dickinson). At least 10,000 events were recorded for each analysis. Data were analyzed using the BD CSampler software (Becton-Dickinson, version 1.0.264.21). Results were recorded as percentage of positivity and ratio of mean fluorescence intensity (rMFI)—the ratio of MFI (PE-conjugated monoclonal antibody) to MFI (appropriate isotype control).

### 2.4. Differentiation Potential

Adipogenic and osteogenic differentiation capacities were assessed by seeding HD-ASC and SSc-ASC at 10,000 cells/cm^2^ on an appropriate surface, including 12-well plates, 24-well plates, and T25 flasks. Adipogenic differentiation and osteogenic differentiation were induced using specific induction media. Adipogenic differentiation was induced using low-glucose DMEM supplemented with 10% FBS, 1 μM dexamethasone (Sigma-Aldrich, Saint-Louis, MO, USA), 0.5 mM 3-isobutyl-1-methylxanthine (Sigma-Aldrich), and 60 μM indomethacine (Sigma-Aldrich). After 14 days of culture, adipogenic differentiation was examined using Oil Red O staining, as previously described [26,27]. Briefly, the cell layer was stained with 1.8 g/L Oil Red O (Sigma-Aldrich) for 30 min. Lipid droplets in the cytoplasm were stained red. Triglyceride accumulation was quantified using a commercially available kit according to the manufacturer’s instructions. Briefly, the cell layer was washed with PBS and incubated with the AdipoRed reagent (Lonza) for 10 min. Fluorescence was measured using a spectrometer (Infinite M200 PRO, Tecan, Männedorf, Switzerland) with an excitation wavelength of 485 nm, an emission wavelength of 572 nm, and a gain of 90. The results were analyzed using i-Control microplate reader software (Tecan, version 3.1.9.0).

After 21 days of culture, osteogenic differentiation was examined using Alizarin Red S (Sigma-Aldrich, Saint-Louis, MO, USA), as previously described [26,27]. Osteogenic differentiation was induced using high-glucose DMEM supplemented with 10% FBS, 0.1 μM dexamethasone, 25 μg/mL L-ascorbic acid (Sigma-Aldrich), and 3 mM NaH_2_PO_4_ (Sigma-Aldrich). Briefly, the cell layer was stained with 2% Alizarin Red S at pH 4.3 (Sigma-Aldrich) for 30 s to 5 min. The staining reaction was stopped with distilled water. Calcium deposits were stained reddish-orange. Calcium content was measured using a commercially available kit (Interchim) following the manufacturer’s instructions. Following differentiation of HD-ASC and SSc-ASC under the tested culture conditions, expression of specific differentiation markers was assessed using quantitative reverse-transcription polymerase chain reaction (qRT-PCR) using the SsoAdvanced Universal SYBR Green Supermix (Bio-Rad, Hercules, CA, USA), a PrimePCR assay (Bio-Rad), and cDNA in a CFX 1000 Touch system (Bio-Rad). Amounts of fatty acid-binding protein 4 (FABP4) and alkaline phosphatase, liver/bone/kidney (ALPL) were normalized to that of glyceraldehyde 3-phosphate dehydrogenase (GAPDH, ΔC_T_ = C_T(gene of interest)_ − C_T(GAPDH)_). 

### 2.5. Senescence-Associated β Galactosidase (SA-β-Gal) Staining

SA-ß-Gal activity was evaluated using the Promokine senescence detection kit (PromoCell) according to the manufacturer’s instructions. The percentage of SA-ß-Gal positive cells was determined in 10 randomly selected microscopic fields (20× magnification) per well. 

### 2.6. Cell Cycle Analysis

Cell cycle analysis was conducted by using a propidium iodide-based flow cytometry method. ASC (100,000 cells/well in six-well culture dishes) were incubated for 48 h in basal medium, harvested, fixed with 70% cold ethanol, and stained with propidium iodide. At least 10,000 events were acquired per sample using a Gallios flow cytometer system and were analyzed using Kaluza analysis software (Beckman Coulter, Kaluza Analysis 2.1).

### 2.7. Analysis of ASC Secretome

ASC were seeded at a density of 100,000 ASC/well in six-well culture dishes with DMEM/F-12 supplemented with 10% FBS for 24 h; the medium was then replaced with 1 mL DMEM supplemented with 1% FBS. The conditioned medium was recovered after 48 h and then centrifuged at 300 *g* for 5 min. Quantification of soluble factors including vascular endothelial growth factor A (VEGF-A), matrix metalloproteinase-2 (MMP-2), tissue inhibitor of metalloproteinase-1 (TIMP-1), hepatocyte growth factor (HGF), and transforming growth factor β1 (TGFβ1) was performed using the Luminex method following the manufacturer’s instructions; 96-well plates were run on a Bio-Plex MAGPIXTM multiplex reader using Bio-Plex Manager software (Bio-Rad, Hercules, CA, USA). Concentrations were calculated based on seven-point standard curves using a five-parametric fit algorithm. Only data that fell within the documented assay range and passed the quality control standards provided by the manufacturer were considered. 

### 2.8. qRT-PCR Analysis

Total RNA was isolated from HD-ASC and SSc-ASC using RNeasy mini kits (Ambion), including a DNase I digestion step to remove genomic DNA. Concentrations were determined using a spectrophotometer (NanoVue, Biochrom). We converted 60 ng RNA to cDNA using a high-capacity cDNA archive kit (Applied Biosystems, Foster, CA, USA). Real-time PCR amplification was performed using Taqman Fast Advanced Master Mix (Applied Biosystems) with pre-designed primers (Applied Biosystems) on a StepOne Real-Time PCR System (Applied Biosystems). The cycling conditions were as follows: 2 min at 50 °C (uracil-N-glycosylase (UNG) incubation), 2 min at 95 °C (polymerase activation), 40 cycles of 1 s at 95 °C (denaturation), and 20 s at 60 °C (annealing/elongation). The primers used for gene-specific amplification are described in Table 1. Threshold cycle (CT) values of technical duplicates were averaged, and relative quantification of all mRNAs of interest was performed based on the 2^−ΔCT^ method for mRNA GAPDH expression. 

### 2.9. Co-Culture Conditions

ASC from HD and SSc patients were co-cultured with dermal fibroblasts (DF) from SSc patients. DF were isolated after enzymatic digestion (collagenase NB 5, 0.25 U/mL for 45 min at 37 °C) of a skin biopsy and seeded at a density of 15,000 cells/cm² dishes in DMEM supplemented with 10% PBS. The medium was replaced with DMEM supplemented with 0.5% FBS 24 h later. On the following day, 288,000 ASC were placed in the top chamber using a transwell insert (Greiner) with a 0.4 µm pore diameter to permit cytokine movement between chambers. After five days of co-culture, Western blot assays and collagen quantification were performed on fibroblast protein lysate.

HD-ASC and SSc-ASC were co-cultured with adult human microvascular dermal endothelial cells (HMVEC-dA; C-12212, Promocell) in an indirect co-culture model using a 0.4 µm pore diameter transwell insert for 24 h. HMVEC-dAs were seeded in six-well culture dishes at a density of 10,500/cm². They were then incubated overnight with endothelial basal cell culture medium 2 (C-22211, Promocell) supplemented with 1% PBS. On the following day, we placed 600,000 ASC in the top chamber using a 0.4 µm pore diameter transwell insert for 24 h. HMVEC-dA were then trypsinazed and tested immediately in angiogenic (tube formation and spheroid) in vitro assays.

### 2.10. Western Blot Analysis

Proteins were extracted from cell layers in six-well-plates and subjected to sodium dodecyl sulfate–polyacrylamide-based discontinuous gel electrophoresis (SDS–PAGE) using NuPAGE 4–12% Bis-Tris Protein Gels (ThermoFisher Scientific, Waltham, MA, USA). SDS-PAGE was performed at 200 V for 60 min followed by protein electroblotting (90 min at 0.5 A and 4 °C) onto nitrocellulose C+ membranes. Immunodetection was performed in Tris-buffered saline supplemented with 0.1% Tween-20 and 3% bovine serum albumin (BSA). Membranes were incubated overnight at 4 °C with primary antibodies to detect alpha smooth muscle actin (α-SMA; A5228, Sigma Aldrich) and GADPH (2118C, Cell Signaling, Danvers, MA, USA). Blots were then incubated with secondary goat anti-rabbit or anti-mouse antibodies (Abcam, Cambridge, UK) for 1 h at room temperature. Immunocomplexes were visualized by enhanced chemiluminescence (ECL) according to the manufacturer’s instructions (Pierce, Rockford, IL, USA). Specific bands were detected using a G-BOX Imaging System (GeneSys, Cambridge, UK), and the optical density (OD) of each band was measured using GeneTools software (Syngene, version 4.03.05.0). All proteins for each panel were assessed on one membrane; thus, GAPDH expression was determined once for all proteins to control for loading. For each sample, differences between proteins of interest and the control loading protein were calculated as relative content and are presented graphically. For statistical analyses, signals were further normalized with the control group (untreated DF).

### 2.11. Collagen Content Quantification

Proteins were extracted from cell layers in six-well-plates after five days of co-culture with ASC based on the Sircol quantitative dye-binding method (Biocolor). Samples were centrifuged at 12,000× *g* for 5 min, and 80 μL of each sample was added to 1 mL Syrius red reagent. Tubes were rocked at room temperature for 30 min and centrifuged at 12,000× *g* for 10 min. Supernatants were discarded, and tubes washed with 750 μL ice-cold salt acid. After another 12,000× *g* centrifugation for 10 min, collagen–dye pellets were suspended in 1 mL alkali reagent. Sample ODs and the standard range of bovine collagen type I concentration were read at 555 nm using a microplate reader (Multiskan Ascent, Thermo Electron, Illkirch, France). Data are expressed as collagen content (µg). 

### 2.12. Tube Formation Assays

HMVEC-dA resulting from co-culture with HD-ASC (*n* = 4) and SSc-ASC (*n* = 6) were loaded at a density of 20,000 cells/well in a µ-slide angiogenesis (81506, IBIDI) system coated with 10 μL 6 mg/mL growth factor-reduced, phenol red-free Matrigel Basement Membrane Matrix (356231, Corning), previously polymerized for 30 min, and maintained in endothelial cell basal medium 2 (EBM2) supplemented with microvascular SingleQuot (EGM2-MV; Lonza) at 37 °C with 5% CO_2_. Capillary-like structures were recorded after 6 h using a video-imaging inverted microscope (DMI8, Leica, Heidelberg, Germany equipped with an I8 incubator at 5× magnification; images were analyzed using Leica Application Suite X software (Las X v. 3.0.2.16120). Mesh and node numbers were quantified using ImageJ software with the Angiogenesis Analyzer plug-in. Each experiment was performed in triplicate.

### 2.13. Spheroid-Based Sprouting Assay

Angiogenic sprouting was analyzed in vitro using the stromal vascular fraction (SVF) in a collagen gel matrix, as previously described [28]. Briefly, HMVEC-dA resulting from co-culture with HD-ASC (*n* = 4) and SSc-ASC (*n* = 6) were suspended in culture medium containing 0.2% (wt/vol) carboxymethylcellulose (M0512, Sigma Aldrich), which was then seeded in non-adherent round-bottom 96-well plates (82.1582.001, Sartstedt), leading to spheroid formation with a defined cell number. After 24 h, spheroids were collected and embedded in collagen gels (354236, rat tail Collagen I, Corning). The spheroid-containing gel was rapidly transferred onto pre-warmed Labtek II slides (NUNC 54534, Thermo Fisher) and allowed to polymerize (30 min); 100 µL of the resulting medium from each co-culture was added to the top of the gel. After 24 h of culture, spheroids were fixed for 30 min in 4% paraformaldehyde at room temperature. After washing and permeabilization for 2 h at 4 °C with PBS supplemented with 0.1% Triton X-100 and 1% BSA, spheroids were immunolabeled overnight at 4 °C with phalloidin coupled to Alexa-647 (A22287, Thermo Fisher Scientific) (1/100), and nuclei were stained with 6-diamidino-2-phenylindole (DAPI) (1/5000) diluted with PBS supplemented with 1% BSA. After washing, we captured a fluorescent optical image stack along the z-axis at 20× magnification using two lasers in sequential mode under a Leica DMI8 microscope (*n* = 10 spheroids per condition). Las X software was used for all image-acquisition procedures. Prior to image measurements, we performed image processing using Huygens Essential deconvolution software (Scientific Volume Imaging, version 18.04 version number) with up to 40 iterations of the classical maximum likelihood estimation algorithm and a theoretical point spread function and automatic background correction. The images were then analyzed using the Sprout Analysis plug-in in the ImageJ Fiji distribution [29] to evaluate total network length and average sprout length.

### 2.14. Statistical Analyses

Statistical analyses were performed using Graph Pad Prism 5 software (GraphPad Software, La Jolla, CA, USA) and IBM SPSS software package (SPSS Statistics for Windows, version 17.0). Quantitative variables are reported as means ± standard deviation (SD) or median with range according to the normality of their distribution. Continuous variables were compared using Student’s *t*-test or the nonparametric Mann–Whitney *U*-test. To compare more than two groups, we used one-way or two-way analysis of variance (ANOVA) followed by a post-hoc Bonferroni test for multiple comparisons. Significance was determined at a level of *p* < 0.05. 

## 3. Results

### 3.1. Patient Characteristics

Baseline characteristics of HD and SSc patients are summarized in Table 2. 

### 3.2. ASC Characterization

#### 3.2.1. Morphology and Phenotype 

**Figure 1:**
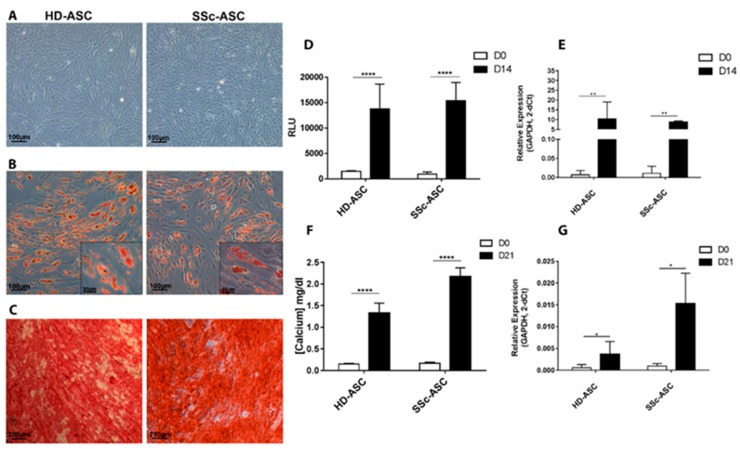
Adipogenic and osteogenic differentiation capacity of ASC from Healthy donors (HD-ASC) and from SSc patients (SSc-ASC). Representative images of HD-ASC and SSc-ASC (**A**) before differentiation (10× magnification), (**B**) after further differentiation into the adipogenic lineage and stained with Oil Red O (10× and 40× magnification), (**C**) and after further differentiation into the osteogenic lineage and stained with Alizarin Red S (5× magnification). (**D**) Accumulation of triglycerides before (D0) and after differentiation into the adipogenic lineage (D14). (**E**) mRNA level of FABP4 before (D0) and after differentiation into the adipogenic (D14), assessed using RT-qPCR. (**F**) Calcium content in cell lysates before (D0) and after differentiation into the osteogenic lineage (D21). (**G**) mRNA level of (ALPL) in HD-ASC and SSc-ASC before (D0) and after differentiation into the osteogenic lineage (D21), assessed using RT-qPCR. Statistical analysis was carried out with a two-way ANOVA test. * *p* < 0.05, ** *p* < 0.01 and **** *p* < 0.0001.

ASC were characterized according to International Society for Cell & Gene Therapy (ISCT) criteria. During cellular expansion, HD-ASC and SSc-ASC were small, fusiform, spindle-shaped, plastic-adherent cells (Figure 1A), a typical morphology for mesenchymal stem/stromal cells [30]. We then analyzed membrane marker expression among HD-ASC and SSc-ASC in accordance with ISCT guidelines [31]. Our results showed that both cell populations expressed typical mesenchymal stromal cell membrane markers (CD13, CD29, CD44, CD73, CD90, and CD105) and did not express hematopoietic markers (CD34 and CD45) or major histocompatibility complex class II markers (HLA-DR). We also verified that culture conditions did not induce expression of co-stimulatory molecules. Both HD-ASC and SSc-ASC were found to be negative for CD40, CD80, and CD86 marker expression (Table 3). Results are presented in percentage of positive cells and ratio of mean fluorescence intensity (rMFI) of each marker to the respective isotype control (means ± SD). Statistical analysis were performed on rMFI results (F-test followed by *t*-test).

#### 3.2.2. Differentiation 

The differentiation potential of HD-ASC and SSc-ASC toward adipogenic and osteogenic lineages after appropriate induction was investigated (Figure 1). Oil Red O staining revealed that HD-ASC and SSc-ASC efficiently differentiated into the adipogenic lineage (Figure 1B). Under adipogenic induction, cells appeared larger and more oval, and large lipid droplet accumulation was observed within the cytoplasm (stained red). Triglyceride quantification showed significant accumulation after 14 days of differentiation regardless of the cellular population studied (Figure 1D). Expression of FABP4, an adipogenic-specific gene, was analyzed using RT-PCR after cell expansion in an FBS + bFGF medium before (day 0, D0) and after (D14) differentiation. FABP4 mRNA expression levels were significantly increased after adipogenic induction at D14 (Figure 1E). 

HD-ASC and SSc-ASC amplified in FBS + bFGF-containing medium showed the ability to differentiate in osteoblasts, as assessed by Alizarin Red S staining (Figure 1C). After three weeks under osteogenic stimuli, we observed the presence of calcium deposits (stained red). The concentration of calcium deposits in the extracellular matrix was significantly higher at D21 than at D0 for HD-ASC and SSc-ASC (Figure 1F). Finally, the expression of ALPL, a key osteogenic-specific gene, was analyzed by RT-PCR before (D0) and after (D21) differentiation (Figure 1G). Levels of ALPL mRNA expression were significantly higher after osteogenic induction at D21 in both cell types. Together, these results show that differentiation potential in adipocytes and osteoblasts was similar in HD-ASC and SSc-ASC. Our results also show that both cellular populations presented typical human mesenchymal stromal cell features.

### 3.3. ASC Senescence Markers 

**Figure 2:**
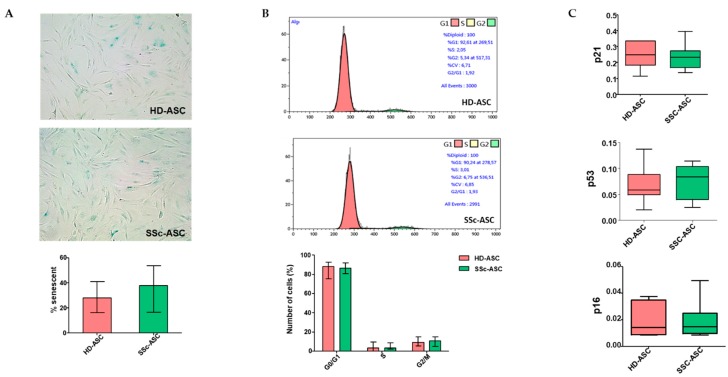
Senescence markers of HD-ASC (*n* = 7) and SSc-ASC (*n* = 7). (**A**) Representative images of βGal staining are shown in upper panel (magnification 20×). The graph (lower panel) represents the percentage of senescent cells determined as the number of cells positive for senescence associated β-Galactosidase (blue cells) relative to the total number of cells in HD-ASC (*n* = 7) and SSc-ASC (*n* = 7). (**B**) Cell cycle analysis by flow cytometry. The DNA content in each cell cycle phase was analyzed by flow cytometry after propidium iodide staining. The representative histograms are from one sample per group (upper panel). The graph (lower panel) represents a mean percentage of cells at different phases of the cell cycle determined by the DNA content for HD-ASC (*n* = 7) and SSc-ASC (*n* = 7). (**C**) Relative mRNA expression of different cell cycle markers was determined by RT-qPCR analysis. For each gene, data are normalized to the housekeeping gene GAPDH.

Using SA-β-Gal staining, we detected no significant difference in senescence percentages between HD-ASC and SSc-ASC (*p* > 0.05; Figure 2A). To confirm the absence of senescence, we analyzed the cell cycle distribution using flow cytometry and found that the percentages of quiescent cells (G0/G1), replicative cells (S), and division cells (M) were similar between HD-ASC and SSc-ASC (*p* > 0.05; Figure 2B). The major pathways controlling senescence converge at the level of activation of the cycle-dependent kinase inhibitor p16^INK4^ or p21^WAF^. Therefore, we assessed the expression of several key cell-cycle markers by quantitative RT-PCR (p16^INK4^, p21^WAF^, and p53). We observed no differences in the expression levels of these markers between the two groups (Figure 2C).

### 3.4. qRT-PCR and Secretome Analyses

**Figure 3:**
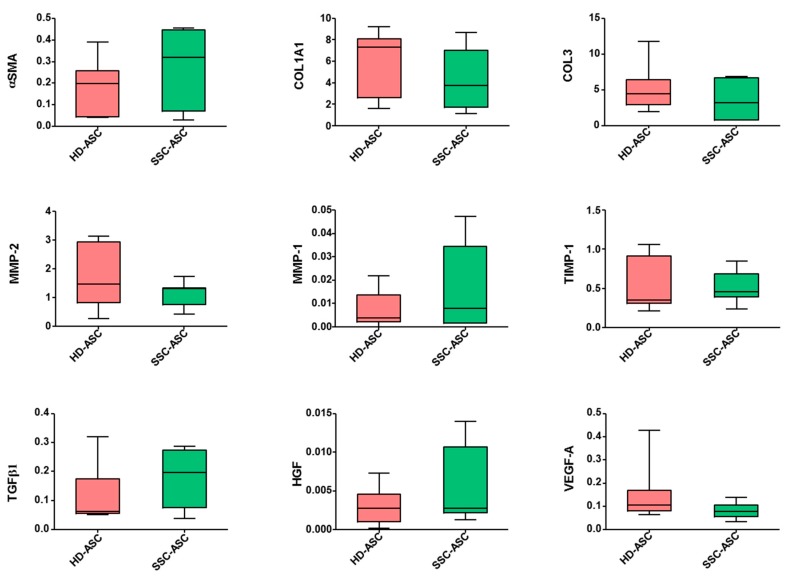
SSc-ASC (*n* = 7) and HD-ASC (*n* = 7) at passage 3–5 were assayed for mRNA expression level of αSMA (alpha smooth muscle actin), COL1A1 (collagen type I alpha 1), COL3 (collagen type 3), MMP-1 and MMP-2 (matrix metalloproteinase 1 and 2), TIMP-1 (tissue inhibitor of metalloproteinase 1), TGFβ1 (transforming growth factor beta 1), HGF (hepatocyte growth factor), and VEGF-A (vascular endothelial growth factor A) by RT-qPCR. GAPDH was measured as an endogeneous control for normalization. For each gene, results are expressed as 2^−ΔCT^. Whiskers represent the median with range (min–max). Statistical analysis was carried out with a Mann–Whitney test.

We then investigated mRNA expression for a set of genes relevant to ASC pro-angiogenic and antifibrotic potential. qRT-PCR analysis showed lower VEGF-A transcript expression levels in SSc-ASC than in HD-ASC (*p* = 0.16). No significant difference was detected in mRNA levels of alpha smooth muscle actin (αSMA), collagen type I alpha 1 (COL1A1), collagen type 3 (COL3), matrix metalloproteinase 1 and 2 (MMP-2, MMP-1), tissue inhibitor of metalloproteinase 1 (TIMP-1), transforming growth factor beta 1 (TGFβ1), or hepatocyte growth factor (HGF) (Figure 3). Similarly, secretome analysis confirmed the trend of lower vascular endothelial growth factor A (VEGF-A) secretion levels in SSc-ASC (*p* = 0.20) and no significant difference among other markers evaluated between the two groups. Table 4 presents each paracrine factor as the mean ± SD (*n* = 3 for each group). Statistical analysis was carried out with a Mann–Whitney test. 

### 3.5. ASC Pro-Angiogenic Paracrine Activity

**Figure 4:**
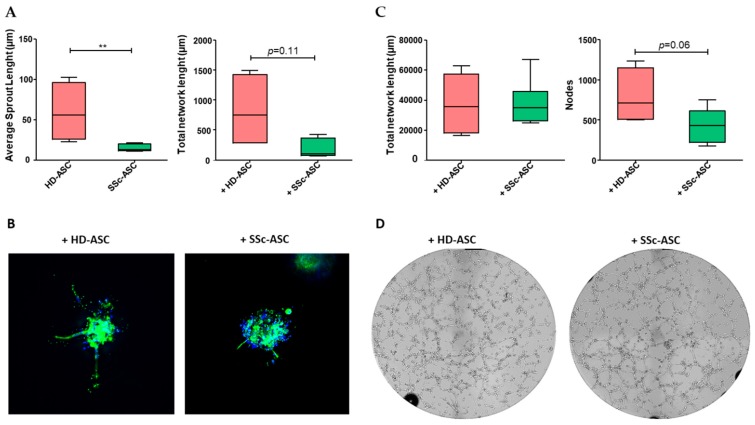
Pro-angiogenic paracrine effect of ASC evaluated in angiogenic in vitro assays with HMVEC-dA co-cultured with HD-ASC (*n* = 4) and SSc-ASC (*n* = 6). (**A**) Quantification of 3D in vitro angiogenesis assay with collagen gel-embedded spheroids (total network length and average sprout length). For each experiment, 10 spheroids were analyzed. Data are median with range (min–max) of HD-ASC (*n* = 4) and SSc-ASC (*n* = 6). (**B**) Representative images of vascular sprout stained for F-actin with phalloidin (red) and nuclei using 6-diamidino-2-phenylindole (DAPI), (blue) (magnification ×20). (**C**) Quantitative analysis of tube formation assays after six hours (total network length and number of nodes). A total of 20,000 HMVEC-da/well were seeded on growth factor-reduced Matrigel. Data are median with range (min–max) of HD-ASC (*n* = 4) and SSc-ASC (*n* = 6) in experiments performed in triplicate. (**D**) Representative images of tube formation assays (10x magnification) six hours after seeding. Statistical analysis was carried out with a Mann–Whitney test. ** *p* < 0.01.

The sprouting ability of HMVEC-dA was evaluated using a 3D spheroid assay performed in vitro after co-culture in the presence of SSc-ASC or HD-ASC (Figure 4A,B). The results indicated that average sprout length was significantly lower in HMVEC-dAs co-cultivated with SSc-ASC compared to those exposed to HD-ASC. The total network length also decreased in response to SSc-ASC, although this difference was not significant. The results of the Matrigel assay indicated that HMVEC-dA-dependent formation of capillary-like structures did not differ significantly between HMVEC-dA stimulated with SSc-ASC and HD-ASC (Figure 4C,D). Together, these findings demonstrate that SSc-ASC maintained a significant paracrine angiogenic effect on endothelial cells, although this effect was slightly smaller than that of HD-ASC.

### 3.6. ASC Antifibrotic Paracrine Potential

**Figure 5:**
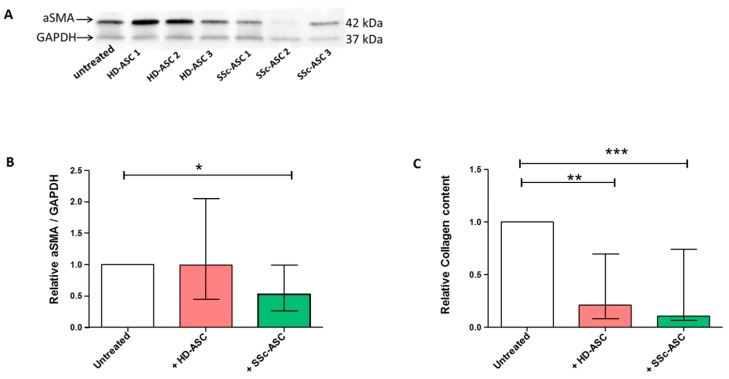
Anti-fibrotic paracrine effect of HD-ASC and SSc-ASC on dermal fibroblasts (DF) from SSc patients. (**A**) The level of αSMA was examined by immunoblot on whole cells lysates from SSc-DF (*n* = 3) after co-culture with HD-ASC (*n* = 4) and SSc-ASC (*n* = 6). Representative experiments for αSMA and GAPDH (loading control) for three independent HD-ASC and SSc-ASC on 1 SSc-DF are shown. (**B**) Protein quantification was represented by bar graphs showing the relative αSMA/GADPH quantification normalized to the untreated SSc-DF condition. (**C**) Histogram displays the relative collagen content in protein lysates of SSc-DF (*n* = 2) after co-culture with HD-ASC (*n* = 4) or SSc-ASC (*n* = 6) normalized to the untreated SSc-DF. Statistical analysis was carried out with a one-way ANOVA test. * *p* < 0.05, ** *p* < 0.01 and **** *p* < 0.0001.

Finally, we evaluated ASC paracrine antifibrotic potential by measuring the expression of the myofibroblast marker αSMA and the collagen content of DF isolated from SSc patients and co-cultured with ASC for five days. Western blot analysis performed on DF protein lysate showed that SSc-ASC secretory activity induced a significant reduction in DF α-SMA expression compared to that of non-treated DF (Figure 5A,B), whereas the effect of HD-ASC was non-significant and heterogeneous among donors. Compared to untreated DF, soluble collagen content was significantly lower in DF stimulated by either SSc-ASC or HD-ASC (Figure 5C). Together, these findings demonstrate the antifibrotic paracrine activity of SSc-ASC in vitro.

## 4. Discussion

ASC-based therapy represents hope for patients suffering from advanced forms of SSc. However, before such therapy can be applied, important aspects about the maintenance of their beneficial biological properties in the autologous context must be completely elucidated. The identification of in vitro functional tests that behave as potency assays, e.g., anticipating clinical results, should permit better selection of patients who can benefit from these innovative therapies. In this study, we demonstrated that SSc-ASC secretory activity promoted significant reduction of fibrosis in vitro. Moreover, the pro-angiogenic potential of ASC isolated from SSc patients was maintained, albeit to a slightly lower extent than observed in HD-ASC. These findings may support further clinical development of autologous ASC to treat SSc patients for whom conventional therapies are no longer an option. 

Although several studies have evaluated the phenotypic and the functional characteristics of bone marrow MSC (BM-MSC) from patients suffering from SSc, few have reported the effects of SSc on MSC extracted from adipose tissue. Our results confirm that, compared with HD-ASC, SSc-ASC display similar expansion potential, adipogenic or osteogenic differentiation potential, and unaltered phenotype. This finding is consistent with those of early studies of BM-MSC and ASC from SSc patients [32,33,34]. However, SSc-ASC have been reported to display multilineage differentiation ability but have failed to sustain a terminally differentiated progeny [25]. Impaired maturation of SSc-ASC in adipocytes or osteocytes was associated with a downregulation of a set of genes involved in cell differentiation. In contrast with our results, Virzì et al. also reported that SSc-ASC expressed CD44, CD29, and CD73 to a lesser extent than those derived from healthy individuals. Based on increased levels of various inflammatory cytokines measured in the SVF, the authors suggested that the pro-inflammatory microenvironment characterizing adipose tissue impairs the differentiation capacity and the phenotype of ASC from SSc patients. Therefore, heterogeneity in the inflammatory status of SSc patients is likely to contribute to discrepancies between studies. The senescence status of MSC is of paramount importance for their therapeutic use, because accelerated senescence impedes in vitro expansion, which is necessary to obtain a sufficient dose of cells and also evokes deleterious changes in the secretome and the MSC biological properties [35]. In contrast to a previous BM-MSC study [36], we demonstrated that SSc-ASC did not exhibit greater senescence than HD-ASC. Senescence is notably associated with decreased activity in sirtuins and is emerging as a pathophysiological contributor to scleroderma [37]; therefore, our results provide further evidence of the benefit of using adipose tissue as an MSC source for therapeutic purposes [38]

Paracrine promotion of angiogenesis is highly likely in SSc, especially when local MSC-based therapies are used to limit vascular manifestations and digital ulcers. We found that paracrine factors produced by SSc-ASC maintained the capacity to stimulate angiogenic activity significantly in microvascular endothelial cells in vitro. This finding is consistent with those of our previous study of adipose-derived stromal vascular fractions from SSc patients, which showed a slight reduction in in vivo neovascularization potential compared to those from HDs [39]. Nevertheless, SSc-ASC paracrine potential was attenuated compared to that of HD-ASC. This difference was observed mainly in spheroid assays, indicating that only the early stages of angiogenesis (sprouting) were altered, whereas the organization of endothelial cells into capillary-like structures in Matrigel, which mainly involves proliferation, migration, and invasion properties, was enhanced to similar extents by SSc-ASC and HD-ASC. We also detected no significant difference in the expression levels of relevant soluble factors involved in ASC angiogenic effects. Nevertheless, global approaches to further characterize ASC secretome would be of interest to confirm these data. These results are consistent with those of another study of ASC enhancement of angiogenic activity in endothelial cells directly co-cultured in an in vitro model; both SSc-ASC and HD-ASC improved the branching indices of endothelial cells [34]. Together, these findings imply that the effect of SSc on the pro-angiogenic capability of ASC does not constitute a major obstacle to using autologous cells for vascular repair/regeneration. However, future studies must identify the specific mechanisms responsible for the weakened ASC-induced paracrine stimulation of endothelial cell sprouting observed in the present study. 

Other research groups have described the potential involvement of organ-resident perivascular MSC-like cells and BM-MSC in myofibroblast generation during fibrosis development associated with SSc [40,41]. However, the transfer of adipose tissue enriched in autologous ASC was recently shown to improve orofacial fibrosis in SSc patients [42]. In the present study, we report the first assessment of paracrine antifibrotic potential of SSc-ASC using indirect co-culture experiments performed with patient-derived DF. Consistent with the presence of antifibrotic factors in SSc-ASC, such as HGF, TIMP-1, and MMP-2, we found that these cells reduced αSMA expression levels and collagen content in DF from SSc patients, demonstrating significant antifibrotic paracrine effects in vitro. These results appear to contrast with recent studies of miRs in silico, which reported a pro-fibrotic ASC signature from SSC patients [23]. Sera from SSc patients have also been shown to favor differentiation of healthy ASC into profibrotic myofibroblast-like cells in vitro [43].

Notably, our approach allowed us to address functional effects of ASC secretomeusing co-culture model with DF from SSc patients. Thus, our results imply that ASC paracrine antifibrotic functions are finely tuned by the disease-associated microenvironment and that ASC are prone to activate compensatory mechanisms to restore local homeostasis. In support of this hypothesis, such adaptability of MSC to microenvironmental factors has already been proposed due to their immunomodulatory properties [44]. 

In conclusion, our results indicate that the paracrine angiogenic properties of autologous ASC are compatible with potential benefits of ASC-based therapy in scleroderma patients. These findings further encourage clinical evaluation of these emerging strategies and provide additional support for the development of cell-free therapies based on ASC secretome. Future studies of the relationship between these paracrine activities evaluated in vitro and corresponding effects of ASC-based therapies in reducing clinical manifestations of SSC will permit the definition of accurate potency assays. Such tests may allow improved selection of putative responder patients to optimize these promising cell-based therapies.

## Figures and Tables

**Table 1:** References of primers used for RT-qPCR experiments.

Gene Abbreviation	Reference
αSMA	Hs00426835_g1
COL1A1	Hs00164004_m1
COL3A1	Hs00943809_m1
MMP-2	Hs01548727_m1
MMP-1	Hs00899658_m1
TIMP-1	Hs01092512_g1
TGFβ1	Hs00998133_m1
HGF	Hs00300159_m1
VEGF-A	Hs00900055_m1
p16^INK4^	Hs00233365_m1
p21^WAF^	Hs00355782_m1
p53	Hs1034249_m1
GAPDH	Hs02786624_g1

αSMA: alpha smooth muscle actin, COL1A1: collagen type I alpha 1, COL3: collagen type 3, MMP-1 and MMP-2: matrix metalloproteinase 1 and 2, TIMP-1: tissue inhibitor of metalloproteinase 1, TGFβ1: transforming growth factor beta 1, HGF: hepatocyte growth factor, VEGF-A: vascular endothelial growth factor A, GAPDH: glycéraldéhyde-3-phosphate déshydrogénase.

**Table 2:** Baseline characteristics of systemic sclerosis (SSc) patients and healthy donors (HD).

	SSc Patients	HD
Gender F/M	7/0	7/0
Age (years, mean ± SD)	45.8 ± 14.2	42.3 ± 13.1
Body mass index (kg/m^2^, mean ± SD)	22.5 ± 2.74	25.9 ± 3.11
Tobacco (yes/no)	1/6	3/4
Arterial hypertension (yes/no)	0/7	0/7
SSc subclassification (Diffuse/Limited)	4/3	NA

NA: Not Applicable, SSc: Systemic Sclerosis, HD: Healthy Donors, F: Female, M: Male.

**Table 3:** Expression of membrane markers by HD-ASC (adipose-derived stem/stromal cells) and SSc-ASC assessed by flow cytometry.

	% of Positive Cells ± SD		rMFI (Ratio of Mean Fluorescence Intensity) ± SD		Statistical Analysis
Membrane Marker	HD-ASC	SSc-ASC	HD-ASC	SSc-ASC	*p*-Value
CD40	0.01 ± 0.00	0.02 ± 0.00	0.97 ± 0.11	0.88 ± 0.01	NS
CD34	0.35 ± 0.00	0.10 ± 0.00	0.97 ± 0.16	0.93 ± 0.02	NS
CD45	0.06 ± 0.00	0.05 ± 0.00	0.94 ± 0.09	0.98 ± 0.03	NS
HLA-DR	0.01 ± 0.00	0.01 ± 0.00	0.93 ± 0.05	0.95 ± 0.02	NS
CD80	0.02 ± 0.00	0.02 ± 0.00	1.14 ± 0.10	1.00 ± 0.02	NS
CD86	0.07 ± 0.00	0.02 ± 0.00	0.95 ± 0.09	1.01 ± 0.03	NS
CD29	78.03 ± 0.14	60.07 ± 0.29	16.76 ± 6.22	15.23 ± 6.99	NS
CD105	80.05 ± 0.13	72.58 ± 0.22	16.86 ± 6.95	22.38 ± 13.65	NS
CD73	98.24 ± 0.00	97.32 ± 0.04	41.34 ± 1.73	46.71 ± 10.99	NS
CD90	99.07 ± 0.01	97.52 ± 0.03	186.35 ± 31.57	171.04 ± 35.40	NS
CD44	98.95 ± 0.01	99.79 ± 0.00	127.78 ± 14.44	161.73 ± 16.12	NS
CD13	99.83 ± 0.00	99.92 ± 0.00	250.33 ± 106.47	352.41 ± 126.74	NS

SSC-ASC: ASC from SSc patients, HD-ASC: ASC from HD, NS: not significant, HLA-DR: human leukocyte antigen-DR, CD: cluster of differentiation, SD: standard deviation.

**Table 4:** Comparative results of secretome analysis produced from SSc-ASC and HD-ASC.

Molecule (pg/mL)	SSc-ASC	HD-ASC	*p*-Value
VEGF-A	33.49 (±12.91)	50.78 (±11.67)	0.20
MMP-2	28,635 (±1435)	25,991 (±3883)	0.70
TIMP-1	39,805 (±48,657)	51,625 (±41,909)	0.51
HGF	10.90 (±1.721)	11.70 (±4.676)	0.82
TGFβ1	667.1 (±72.94)	620.3 (±64.06)	0.70
